# The Effect of Fosinopril on Achilles Tendon Healing in a Rat Model

**DOI:** 10.3390/ijms27208969

**Published:** 2026-10-09

**Authors:** Şemsettin Can Asar, Mehmet Sabri Balık, Levent Tümkaya, Tolga Mercantepe, Atilla Topçu, Rıfat Şahin

**Affiliations:** 1 Department of Orthopedics and Traumatology, Faculty of Medicine, Recep Tayyip Erdogan University, 53100 Rize, Turkey; 2 Department of Histology and Embryology, Faculty of Medicine, Ondokuz Mayıs University, 55139 Samsun, Turkey; 3 Department of Histology and Embryology, Faculty of Medicine, Recep Tayyip Erdogan University, 53100 Rize, Turkey; 4 Department of Pharmacology, Faculty of Medicine, Recep Tayyip Erdogan University, 53100 Rize, Turkey

**Keywords:** fosinopril, tendon healing, rat, Achilles tendon, ACE inhibitors

## Abstract

In addition to their antihypertensive effects, angiotensin converting enzyme (ACE) inhibitors possess endothelial protective properties. It has been shown that ACE inhibitors reduce fibrosis and adhesion during the tendon healing process by suppressing angiotensin II. In this experimental study, we aimed to investigate the effect of fosinopril, a commonly used ACE inhibitor in the treatment of hypertension, on Achilles tendon healing in rats. A total of 80 Sprague–Dawley rats were used and divided into four groups: healthy control group (Group 1), Achilles tendon rupture and repair group (Group 2), Achilles tendon rupture and repair with fosinopril administration (Group 3), and healthy rats treated with fosinopril only (Group 4). At the end of the experiment, rats were sacrificed at the 1st, 2nd, and 4th weeks, and the groups were compared using biochemical and histological methods. For statistical analysis, data distribution was assessed for normality. Group comparisons were conducted using one-way ANOVA or the Kruskal–Wallis test as appropriate, followed by post hoc pairwise comparisons with correction for multiple testing. According to the biochemical analysis, the levels of TBARS (a marker of oxidative stress) were significantly lower, and total thiol levels (an antioxidant marker) were significantly higher in the fosinopril-treated groups compared to those without fosinopril treatment. In histopathological evaluations using a damage scoring system for edema, hemorrhage, and inflammation, scores were lower in the fosinopril-treated groups. Immunohistochemical analyses revealed that the expression levels of VEGF, collagen I, and collagen III were lower in the fosinopril-treated groups compared to the untreated groups at the same time points. This study demonstrates that fosinopril exerts antioxidant effects during the Achilles tendon healing process and provides favorable histopathological findings during the evaluated period of tendon healing.

## 1. Introduction

Tendon injuries have become increasingly common orthopedic issues today, partly due to lifestyle changes brought about by industrialization and a growing interest in sports activities. Among the largest and most resilient tendons in the human body, the Achilles tendon is also the most frequently affected by various pathologies [1]. Like other tendon injuries, Achilles tendon ruptures can be treated either conservatively or surgically [2]. However, conservative treatments may not always restore severely damaged tendons to their pre-injury functional state. Therefore, surgical treatment methods are more commonly preferred in severe acute injuries such as complete acute tendon ruptures [3,4].

Hypertension is a major public health concern due to its increasing global prevalence. It often requires long-term medical management and can lead to serious chronic complications.

Angiotensin-converting enzyme (ACE) inhibitors are widely used, effective, and reliable medications in the treatment of hypertension [5,6]. In addition to their antihypertensive effects, ACE inhibitors have been reported to exert several pleiotropic biological effects, including endothelial protection, anti-inflammatory activity, antioxidant effects, and modulation of extracellular matrix remodeling. These effects are largely mediated through suppression of angiotensin II-induced oxidative stress and inflammatory signaling pathways. Recent experimental studies have also demonstrated that inhibition of the renin–angiotensin system may reduce fibrosis and adhesion formation in injured tendon tissue by limiting fibroblast activation and collagen overproduction [7,8,9]. These findings suggest that ACE inhibitors may influence the biological environment of tendon healing beyond their cardiovascular effects.

Traditional ACE inhibitors are primarily excreted through the kidneys; therefore, dose adjustments may be necessary in elderly patients with impaired renal function or elevated serum creatinine levels [10,11]. Fosinopril, a newer generation ACE inhibitor, follows a balanced elimination route, with increased hepatic excretion as renal function declines. This characteristic eliminates the need for dose adjustment in patients with renal impairment [12,13,14].

Studies investigating the effects of ACE inhibitors on tendon healing are limited in the literature [15]. Although the renin–angiotensin system has been extensively studied in cardiovascular and renal diseases, its role in musculoskeletal tissue repair has only recently gained attention. Experimental studies have shown that ACE inhibitor therapy may reduce postoperative tendon adhesion formation, decrease inflammatory cell infiltration, and modulate collagen synthesis during tendon healing [15,16]. However, data specifically evaluating the potential effects of fosinopril on tendon regeneration remain scarce. Despite the growing interest in the biological effects of the renin–angiotensin system on tissue remodeling and fibrosis, the influence of ACE inhibitors on the tendon healing process has not been sufficiently clarified.

Therefore, the present study aimed to investigate the effects of fosinopril on the tendon healing process using biochemical and histological parameters following experimentally induced Achilles tendon injury in rats. In particular, oxidative stress status and antioxidant capacity were evaluated through thiobarbituric acid reactive substances (TBARS) and total thiol measurements, while histopathological and immunohistochemical analyses were performed to assess inflammation, collagen organization, and angiogenic activity. Furthermore, by comparing injured tendon tissue with healthy controls, this study sought to clarify the potential role of fosinopril in regulating key biological processes involved in tendon repair, including oxidative stress, inflammation, angiogenesis, and extracellular matrix remodeling.

## 2. Results

### 2.1. Biochemical Analyses

Serum TBARS levels differed significantly among the study groups (*p* = 0.001). The highest oxidative stress was observed in the Repair—Day 7 group (14.28 ± 7.00 nmol/mL). Fosinopril administration, both in the Repair + Fosinopril (9.20 ± 2.37 nmol/mL) and Drug-only (8.67 ± 2.41 nmol/mL) groups, significantly reduced TBARS levels at the same time point. By Day 28, TBARS levels in all groups approached baseline values observed in the Healthy Control group (7.14 ± 0.55 nmol/mL), supporting the antioxidant effect of fosinopril during tendon healing (Table 1).

### 2.2. Serum Total Thiol Levels

Serum total thiol levels differed significantly among groups (*p* = 0.002). The lowest antioxidant capacity was observed in Repair—Day 7 and Repair—Day 14 groups (198 [82] and 186 [135] µmol/L, respectively). Fosinopril administration increased thiol levels in both Repair + Fosinopril and Drug-only groups at all time points. By Day 28, the Drug-only group showed the highest thiol levels (360 [114] µmol/L), exceeding baseline values in Healthy Controls. These findings indicate that fosinopril enhances systemic antioxidant defenses during tendon healing (Table 2). Pairwise comparisons were performed using the Mann–Whitney U test with Bonferroni correction. Significant differences are identified in Table 2 according to the specific reference group and time point indicated by each symbol.

## 3. Histological and Immunohistochemical Analyses

### 3.1. Histopathological Findings

When sections of tendon tissue were examined under a light microscope, only the drug-treated 7th, 14th, and 28th day groups had collagen fibrils with a typical structure in their tendon tissues (Figure 1A–F, Table 3, HHS scores: 0 (0–1), 0 (0–1), and 1 (0–1), respectively; *p <* 0.001). In contrast, the repair group showed widespread edematous areas, neutrophil infiltration, and hemorrhagic areas in the lesion regions of the tendon sections on Day 7 (Figure 1G–I, Table 3, HHS score 6.5 (5–7)). We observed reduced edematous areas, inflammation, and hemorrhage in the tendon tissue on Days 14 and 28 compared to the Day 7 drug-only group (Figure 1G–O, Table 3, HHS scores 6.5 (5–7), 5.5 (5–6), and 5 (4–5.5), respectively; *p <* 0.001). The repair + drug combination treatment resulted in a decrease in, polymorphonuclear leukocyte (PMNL) inflammation and hemorrhagic findings, especially in edematous areas, in the tendon tissues on days 7, 14, and 28 compared to the repair groups on days 7, 14, and 28 (Figure 1P–X, Table 3, HHS scores were 4 (3–4), 4 (3–4), and 2 (2–3), respectively; *p* < 0.001). In particular, the repair + drug combination treatment on day 7 showed a remarkable decrease in PMNL inflammation and hemorrhagic findings, especially in edematous areas, in the tendon tissues compared to the repair group on day 7 (Figure 1M–R, Table 3, *p* < 0.001). In the repair + drug combination group on day 14, we found a reduction in PMNL inflammation and hemorrhage findings, including edematous areas, compared to the repair group on day 14 (Figure 1M–O,S–U, Table 3, *p* < 0.001). Similarly, in the repair + drug combination group on day 28, we found a reduction in PMNL inflammation and hemorrhage findings, including edematous areas, compared to the repair group on day 28 (Figure 1P–R,V–X, Table 3, *p* < 0.001).

### 3.2. Immunohistochemical Analysis

#### 3.2.1. Collagen I (Col1a3) Primary Antibody

When sections of tendon tissue incubated with collagen I primary antibody were examined under a light microscope, we found that the tendon tissue in the control group on days 28 was normal (Figure 2A, Table 4, Collagen I positivity scores were 0 (0–0), 0 (0–0), and 0 (0–0), respectively). Similarly, we observed that only the drug-treated groups on days 7, 14, and 28 were negative for collagen I immunodeficiency in their tendon tissues (Figure 2B–D, Table 4, Collagen I positivity scores were 0 (0–0), 0 (0–0.5), and 0 (0–0), respectively). In contrast, areas showing intense collagen I positivity were present in the day 7 and day 14 tendon sections of the repair treatment group (Figure 2E,F, Table 4, collagen I positivity score 2 (2–3); *p* < 0.001). We observed decreased collagen I positivity in the day 28 tendon tissue of the repair treatment group compared to the day 7 drug-only group (Figure 2B–G, Table 4, collagen I positivity scores, respectively: 2 (2–3), 1.5 (1–2); *p* = 0.007). In the repair + drug combination treatment group, collagen I positivity decreased on days 7, 14, and 28 compared to the repair group (Figure 2D–J, Table 4, collagen I positivity scores were 1 (1–1), 1 (0–1), and 0 (0–1), respectively; *p* < 0.001; *p* = 0.002; *p* = 0.002). In particular, the decrease in collagen I positivity in the repair + drug combination treatment group on day 7 was noteworthy compared to the repair group on day 7 (Figure 2D,H, Table 4, *p* < 0.001). Similarly, we found a decrease in collagen I positivity in the repair + drug combination treatment group on day 14 compared to the repair group on day 14 (Figure 2D,I, Table 4, *p* = 0.002). Similarly, we found that the repair + drug combination treatment group showed decreased collagen I positivity, including edematous areas, in tendon tissue on day 28 compared to the repair treatment group on day 28 (Figure 2G,J, Table 4, *p* = 0.002).

#### 3.2.2. Collagen III (Col3a1) Primary Antibody

When sections of tendon tissue incubated with collagen III primary antibody were examined under a light microscope, we found that the tendon tissue in the control group on days 28 was normal (Figure 3A, Table 4, Collagen III positivity scores were 0 (0–0), 0 (0–0), and 0 (0–0), respectively). Similarly, we observed that only in the drug-treated groups on days 7, 14, and 28, the tendon tissues were negative for collagen III (Figure 3B–D, Table 4, Collagen III positivity scores were 0 (0–0), 0 (0–0), and 0 (0–0), respectively). In contrast, repair treatment group 7 and day 14 tendon sections showed areas with high Collagen III positivity (Figure 3E,F, Table 4, Collagen III positivity score 3 (3–3); *p* < 0.001). We observed decreased Collagen III positivity in the repair-only group compared to the drug-only group (Figure 3B–I, Table 4, Collagen I positivity scores 3 (3–3), 1.5 (1–2), respectively; *p* = 0.007). In the repair + drug combination treatment group, collagen III positivity decreased on days 7, 14, and 28 compared to the repair group (Figure 3E–G, Table 4, collagen III positivity scores were 1 (1–1), 1 (0–1), and 0 (0–1), respectively; *p* < 0.001; *p* = 0.002; *p* = 0.002). In particular, the decrease in collagen III positivity in the repair + drug combination treatment group on day 7 was noteworthy compared to the repair group on day 7 (Figure 3E,H, Table 4, *p* < 0.001). Similarly, we found a decrease in collagen III positivity in the repair + drug combination treatment group on day 14 compared to the repair group on day 14 (Figure 3F,I, Table 4, *p* = 0.002). Similarly, we found that the repair + drug combination treatment group showed a decrease in collagen III positivity, including edematous areas, in tendon tissue on day 28 compared to the repair treatment group on day 28 (Figure 3G,J, Table 4, *p* = 0.002).

#### 3.2.3. VEGF Primary Antibody

When sections of tendon tissue incubated with VEGF primary antibody were examined under a light microscope, we found that the tendon tissue in the control group on days 28 was normal (Figure 4A, Table 4, VEGF positivity scores: 0 (0–0), 0 (0–0), and 0 (0–0), respectively). Similarly, we observed VEGF immunonegative only in the tendon tissues of the drug-treated groups on days 7, 14, and 28 (Figure 4C,D, Table 4, VEGF positivity scores: 0 (0–0), 0 (0–0.5), and 0 (0–0), respectively). In contrast, areas showing intense VEGF positivity were present in the tendon sections of the repair group on day 7 and day 14 (Figure 4E, Table 4, VEGF positivity score 2 (2–3); *p* < 0.001). We observed a decrease in VEGF positivity in the tendon tissue on day 28 in the repair-only group compared to the drug-only group on day 7 (Figure 4B–G, Table 4, VEGF positivity scores were 2 (2–3), 1.5 (1–2), respectively; *p* = 0.007). In the repair + drug combined treatment, there was a decrease in VEGF positivity in the tendon tissues on days 7, 14, and 28 compared to the repair-only groups on days 7, 14, and 28 (Figure 4E–J, Table 4, VEGF positivity scores were 1 (1–1), 1 (0–1), and 0 (0–1), respectively; *p* < 0.001; *p* = 0.002; *p* = 0.002). Specifically, the decrease in VEGF positivity in the tendon tissue of the repair + drug combination group on day 7 was noteworthy compared to the repair-only group on day 7 (Figure 4E–H, Table 4, *p* < 0.001). Similarly, we found a decrease in VEGF positivity in the tendon tissue of the repair + drug combination group on day 14 compared to the repair-only group on day 14 (Figure 4F,I, Table 4, *p* = 0.002). Likewise, we found a decrease in VEGF positivity, including edematous areas, in the tendon tissue of the repair + drug combination group on day 28 compared to the repair-only group on day 28 (Figure 4G–J, Table 4, *p* = 0.002).

Histological evaluation of the control group tendon sections showed a typical tendon structure with normally arranged parallel collagen fibers. Likewise, in the groups that received only fosinopril treatment on days 7, 14, and 28, the tendon tissue maintained a structure similar to normal anatomy, with parallel collagen fibrils preserved.

In the group that underwent only surgical repair (day 7), prominent edematous areas were observed at the incision site. Additionally, neutrophil-dominant inflammation and hemorrhagic findings were present. Degenerated collagen fibers were also widespread in this group. By day 28 in the same group, edema had substantially regressed, and collagen fiber alignment had become more organized (*p* < 0.001).

In the groups that received fosinopril treatment alone, edema, inflammation, and hemorrhage were reduced on days 14 and 28 compared to day 7. In the group that received both surgical repair and fosinopril (day 28), edema, PMNL infiltration, and hemorrhagic areas were markedly reduced in the lesion area compared to the control group. In contrast, newly synthesized, well-organized parallel collagen fibrils were prevalent. This difference was statistically significant (*p* < 0.001) (Figure 1).

In the tendon tissues of the groups treated with both surgical repair and fosinopril on days 7, 14, and 28, a notable reduction in edematous areas, PMNL infiltration, and hemorrhage was observed compared to the groups that underwent surgical repair alone. Additionally, newly synthesized collagen fibrils were widespread, and collagen fibers exhibited a parallel organization. This difference was statistically significant (*p* < 0.001).

In the immunohistochemical analyses, tendon tissue sections incubated with primary antibodies against VEGF, collagen type I, and collagen type III were evaluated under a light microscope. The tendon tissues from the healthy control group preserved their normal histological architecture (Figure 2, Figure 3 and Figure 4).

In the tendon tissue sections from the Day 7 and Day 14 repair-only groups, areas with intense positive expression of VEGF were considered an indicator of neovascularization and remodeling. The key structural proteins, collagen types I and III, which are essential for tendon regeneration, were observed (*p* < 0.001). In contrast, in the groups that received both repair and fosinopril on days 7, 14, and 28, a statistically significant reduction in the expression levels of VEGF, collagen type I, and collagen type III was detected compared to the corresponding repair-only groups (*p* < 0.001, *p* = 0.002, and *p* = 0.002, respectively) (Table 4).

Further statistical comparisons revealed significant differences between the healthy control group and the repair-only groups on days 7, 14, and 28 (*p* < 0.001). Similarly, significant differences were found between the drug-only groups and both the repair and repair + drug groups (*p* = 0.001–0.046). Moreover, statistically significant differences were observed between the Day 7 repair group and the Day 7 repair + drug group (*p* = 0.001), the Day 14 repair group and the Day 14 repair + drug group (*p* = 0.002), and the Day 28 repair group and the Day 28 repair + drug group (*p* = 0.001–0.002) (Table 4, Figure 5).

## 4. Discussion

In this experimental study, the effects of fosinopril, a widely used ACE inhibitor in the treatment of hypertension, on the healing process after surgically repaired Achilles tendon rupture were evaluated at both biochemical and histopathological levels. Our findings demonstrate that systemic administration of fosinopril reduces oxidative stress markers, enhances antioxidant capacity, and significantly decreases edema, inflammation, and hemorrhage from a histopathological perspective. Furthermore, immunohistochemical analyses revealed significantly lower expression levels of VEGF, collagen type I, and collagen type III in the fosinopril-treated groups. These results suggest that fosinopril may exert a modulatory effect on tendon healing and potentially limit excessive scar tissue formation.

The renin–angiotensin system has recently been recognized as an important regulator of tissue remodeling and fibrosis in multiple organs. Angiotensin II stimulates fibroblast proliferation, collagen synthesis, and inflammatory mediator release during tissue injury. Consequently, inhibition of angiotensin II production through ACE inhibitors may attenuate fibrotic responses and regulate extracellular matrix remodeling during tissue repair. Experimental studies have demonstrated that ACE inhibitor therapy may modulate collagen synthesis and extracellular matrix remodeling. In particular, in vitro studies have demonstrated that ACE inhibitors can inhibit the conversion of procollagen to collagen [17]. In addition, experimental tendon repair models suggest that ACE inhibition may reduce adhesion formation and excessive collagen accumulation; however, the overall effects on tendon healing remain incompletely understood [15]. The healing process of tendon ruptures is generally slow. Even when surgical treatment is applied, tendons often fail to fully regain their pre-injury biological and mechanical properties [16]. This biomechanical weakness primarily stems from disorganized collagen fibers, alterations in extracellular matrix components, and scar tissue formation [18]. As a result, significant risks such as adhesions, joint contractures, and even re-injury may arise during the recovery period [19].

Animals such as dogs, pigs, rabbits, and rats are frequently used in experimental studies on tendon healing [20,21,22]. In the selection of experimental animals, factors such as the type of injury to be induced, the technique to be applied, and the similarity of the relevant tissue to human tissue play a crucial role [22]. Rats, in particular, show a high degree of genetic and histological similarity to human tissues [20]. Additionally, rats are commonly preferred in the literature to ensure comparability with previous studies [21]. For these reasons, rats were chosen as the model organism in our study.

Both systemic and local administration approaches for pharmacological agents targeting tendon healing have been investigated in experimental tendon models [15,23,24]. While these findings suggest a potential role for ACE inhibitors in tendon healing, the optimal route of administration and the specific effects of individual ACE inhibitors remain insufficiently explored. Therefore, the present study aimed to further evaluate the effects of oral fosinopril administration on the biochemical and histological parameters of tendon repair. In addition, imaging-based techniques such as ultrasound may offer complementary information regarding tendon structure, vascularization, and tissue organization during the healing process. Integration of these techniques could enhance the assessment of tendon repair in future experimental studies [25]. Although the present study was conducted in an animal model, translational approaches involving minimally invasive ultrasound-guided interventions have increasingly been applied in the clinical management of tendinopathy. For example, ultrasound-guided percutaneous electrolysis has been reported to target pathological tendon tissue and stimulate tissue remodeling, thereby providing clinically relevant insight into the biological mechanisms involved in tendon healing [26]. In the biochemical analyses of blood samples collected from the rats, TBARS and total thiol group levels were measured to assess oxidative stress. TBARS are by-products of lipid peroxidation and are widely used as indicators of oxidative damage [27]. Thiol groups, on the other hand, are compounds that play a key role in the antioxidant defense system. As expected, the group with the highest TBARS levels was the Day 7 repair-only group. The significantly lower TBARS levels observed in the fosinopril-treated groups at the same time point suggest a potential link between ACE inhibitors and antioxidant mechanisms. Previous experimental studies have reported that ACE inhibitors reduce angiotensin II-mediated oxidative stress by suppressing NADPH oxidase activity and superoxide production in endothelial and inflammatory cells. On the basis of these previous reports, ACE inhibition could potentially contribute to improved redox balance and antioxidant defense during tissue healing [14,27]. The highest total thiol levels were found in the Day 28 group that received fosinopril only. When comparing the repair and repair + drug groups at the same time points, thiol levels were significantly higher in the repair + drug groups. These findings are consistent with previous studies indicating that ACE inhibitors may reduce angiotensin II levels, thereby suppressing superoxide production in the endothelium and enhancing antioxidant effects.

In the present study, tendon healing was evaluated histopathologically using a semi-quantitative scoring system based on edema, inflammation, and hemorrhage. These parameters (edema, inflammation, and hemorrhage) are commonly used indicators for the histopathological assessment of tendon healing. In our study, when comparing the repair and repair + fosinopril groups at the same time points, edema levels were found to be lower in the repair + fosinopril group. A previous study has shown that ACE inhibitors reduce edema in scar Tissue [7]. The lowest level of inflammation was observed in the Day 7 repair + drug group, which may be attributed to the ability of ACE inhibitors to suppress the proinflammatory effects of angiotensin II. The most prominent hemorrhagic findings were observed in the Day 7 repair-only group, while the lowest levels of hemorrhage were detected in the Day 14 and Day 28 fosinopril-treated groups. By suppressing the inflammatory actions of angiotensin II, it appears that hemorrhagic changes occurring during the early phase of inflammation are also reduced. These findings suggest that fosinopril may accelerate the tendon healing process by reducing edema, inflammation, and hemorrhage.

In immunohistochemical assessments, VEGF expression was markedly lower in the repair + fosinopril groups compared to the corresponding repair-only groups at the same point. VEGF is a critical mediator of angiogenesis and plays an essential role in the early stages of tissue repair. However, excessive VEGF expression can contribute to pathological neovascularization and fibrotic remodeling. Experimental studies investigating the renin–angiotensin system have demonstrated that ACE inhibition can modulate VEGF expression and angiogenic signaling pathways in various tissues [14,28]. Additionally, collagen type I and type III expression levels were reduced in the repair + fosinopril groups relative to the repair-only groups. This reduction may be associated with the inhibitory effects of fosinopril on fibroblast activation and collagen synthesis through suppression of angiotensin II-mediated signaling pathways. By decreasing oxidative stress and inflammatory responses, ACE inhibition may limit excessive extracellular matrix accumulation and fibrotic scar formation during tendon healing.

Angiotensin II has been shown to stimulate fibroblast proliferation, collagen synthesis, and profibrotic signaling, particularly via activation of transforming growth factor-β and oxidative stress-related mechanisms. Accordingly, ACE inhibition may suppress these profibrotic pathways and regulate extracellular matrix remodeling during tissue repair [8,9]. Moreover, experimental studies indicate that blockade of the renin–angiotensin system can reduce adhesion formation and fibrotic tissue development following tendon injury [15]. These findings support the hypothesis that ACE inhibition may influence both angiogenic activity and collagen turnover during tendon healing. However, angiotensin II levels, TGF-β signaling, and NADPH oxidase activity were not directly assessed in the present study; therefore, these pathways are discussed only as literature-supported potential mechanisms and should not be interpreted as mechanistically demonstrated mediators of the observed effects of fosinopril.

An important distinction should be made between the physiological collagen synthesis required for normal tendon repair and excessive or prolonged collagen deposition that may contribute to fibrosis and scar formation. Collagen types I and III are essential components of tendon healing, and adequate collagen production is necessary for restoration of the extracellular matrix. Therefore, the lower collagen I and III immunoreactivity observed in the repair + fosinopril groups should not be interpreted as evidence that suppression of collagen expression is inherently beneficial or that lower collagen expression necessarily represents better healing. Notably, histopathological examination in the present study demonstrated newly synthesized collagen fibrils with a more organized parallel arrangement in the fosinopril-treated repair groups, despite the lower semi-quantitative collagen I and III immunoreactivity. This finding may suggest modulation rather than complete suppression of the reparative collagen response. Nevertheless, excessive down-regulation of collagen synthesis could theoretically impair extracellular matrix formation and compromise tendon strength, particularly during the early phases of repair. Because collagen expression was assessed semi-quantitatively and biomechanical testing was not performed, the present study cannot determine whether the observed reduction represents favorable regulation of excessive collagen deposition or potentially insufficient collagen synthesis. Thus, the hypothesis that fosinopril limits excessive scar formation while preserving adequate tendon repair requires confirmation through quantitative collagen assessment, biomechanical and functional testing, and longer-term studies.

The effects of ACE inhibitors on collagen synthesis remain a subject of debate in the literature. Some studies have reported that ACE inhibitors can inhibit the conversion of procollagen to collagen [17]. In contrast, other studies have shown that ACE inhibitors can increase collagen type III synthesis, reflecting upregulation of collagen turnover in certain clinical and experimental settings [29]. In the present study, expression levels of VEGF, collagen type I, and collagen type III were lower in the repair + fosinopril groups compared to the repair-only groups. Some studies have reported that fosinopril reduces the expression of VEGF and collagen types I and III in the long term [28]. Considering the tissue-reparative effects of fosinopril, our findings suggest that further studies focusing on the early stages of tendon injury (the initial days and weeks) are warranted to support and expand upon the results of this study.

## 5. Materials and Methods

This prospective experimental study was conducted following the approval of the Institutional Animal Care and Use Committee of a tertiary university hospital (Decision No: 2020/50, dated 31 December 2020). A total of 80 adult female Sprague–Dawley rats, each weighing approximately 280–340 ± 15 g, were used in the study. All animals were housed and fed under sterile conditions in an experimental animal facility with a 12 h light/12 h dark cycle, 55–60% humidity, and a controlled room temperature of 22 ± 3 °C.

Randomization and experimental allocation: Following a one-week acclimatization period, all animals were randomly allocated to the experimental groups using a computer-generated randomization list. Each rat was assigned an identification number prior to group allocation to ensure unbiased distribution among the groups and subgroups. Animals were housed under identical environmental conditions, and cage distribution was arranged to minimize potential cage-related or environmental effects.

The rationale behind the group classification was as follows: Group 1 was designated as the healthy control group. Groups 2 and 3 included rats that underwent Achilles tendon rupture followed by surgical repair. In addition, Group 3 received fosinopril treatment. Group 4 consisted of healthy rats treated with fosinopril only, without any surgical intervention. All groups were sacrificed at specific time points (weeks 1, 2, and 4) to evaluate the effects of the interventions over time (Table 5).

Thanks to this study design, it was possible to compare: the effect of surgical intervention (Group 2 vs. Group 1), the effect of pharmacological treatment (Group 3 vs. Group 2), and the effect of the drug on healthy subjects (Group 4 vs. Group 1).

Fosinopril sodium (Monopril 10 mg, Deva İlaç Sanayi, Istanbul, Turkey), stored under appropriate conditions, was administered orally at a dose of 5 mg/kg. Previous experimental rat studies have shown that fosinopril is pharmacologically active and exhibits tissue-protective effects [30,31]. A total of 48 rats, excluding those in the healthy control and tendon rupture + repair-only groups, received 1 cc of the drug daily via an orogastric cannula. The administration was performed at the same time every day throughout the study period. After two subgroups were sacrificed at week 2, fosinopril treatment continued in the remaining 32 rats at the same dosage.

All rats in Groups 3 and 4 received fosinopril daily, starting immediately after surgical repair for Group 3 or at the corresponding time for healthy rats in Group 4, ensuring consistency across subgroups. Rats in Groups 1 and 2 did not receive any drug treatment, preserving their roles as untreated controls for both surgical and pharmacological comparisons.

For rats sacrificed at week 2, fosinopril treatment was continued in the remaining animals until their designated sacrifice time points (week 4), maintaining uniform exposure duration within each experimental subgroup. This design ensures that comparisons between treated and untreated animals, as well as between healthy and injured subjects, remain valid and interpretable.

### 5.1. Sample Size Consideration

The number of animals per subgroup (*n* = 8) was determined based on previously published experimental tendon healing studies reporting measurable biochemical and histopathological differences using comparable group sizes. In addition, ethical considerations in animal research, particularly the 3R principles (Replacement, Reduction, and Refinement), were taken into account to minimize unnecessary animal use while maintaining the scientific validity of the experiment.

During surgical procedures, anesthesia was achieved via intraperitoneal injection of 50 mg/kg Ketamine HCl (Ketasol 10%, Richter Pharma AG, Wels, Austria) and 5 mg/kg Xylazine HCl (Rompun 2%, Bayer İlaç Sanayi, Istanbul, Turkey), mixed in the same syringe (1 cc ketamine + 2 cc Rompun). All surgeries were performed by the same surgeon (Figure 6).

### 5.2. Blood Sample Collection and Biochemical Analysis

Blood samples were collected from the rats into anticoagulant-free tubes and left at room temperature for approximately one hour to allow for fibrin formation. The samples were then centrifuged at 1000× *g* for 15 min at a temperature of 2–8 °C. The resulting serum supernatants were transferred into Eppendorf tubes (Hamburg, Germany)and stored at −80 °C until the day of analysis.

Histological and immunohistochemical assessments were performed by two independent evaluators blinded to the group allocations. In addition, all personnel involved in blood collection, serum preparation, and biochemical analyses were blinded to the experimental groups. Although the surgeon necessarily performed the surgical repair, personnel administering fosinopril were blinded to the study hypotheses and expected outcomes, ensuring that drug handling did not introduce systematic bias. These measures minimize detection and performance bias, particularly in semi-quantitative histological scoring. Biochemical and histopathological analyses were performed by investigators who were blinded to the group allocation in order to minimize potential assessment bias.

Oxidative stress levels in serum samples were evaluated by TBARS using a modified method based on the procedure described by Draper and Hadley [32]. In this assay, serum samples were reacted with thiobarbituric acid under acidic and high-temperature conditions to form a colored malondialdehyde–TBA complex. The absorbance of the resulting chromogen was measured spectrophotometrically at 532 nm using a UV–visible spectrophotometer. TBARS concentrations were calculated using a calibration curve prepared with standard malondialdehyde solutions and were expressed as nmol/mL serum. All biochemical analyses were performed in duplicate to improve analytical reliability.

To assess antioxidant capacity, total thiol groups were measured using Ellman’s reagent, based on the detection of free SH groups. Briefly, serum samples were incubated with 5,5′-dithiobis-(2-nitrobenzoic acid) (DTNB), and the formation of the yellow-colored 5-thio-2-nitrobenzoic acid product was measured spectrophotometrically at 412 nm. Quantification was performed using a standard curve generated with reduced glutathione, and the results were expressed as µmol/L. All measurements were conducted in duplicate, and the mean value was used for statistical analysis.

Quality control procedures included the use of standard calibration curves and repeated measurements. Intra-assay variability was monitored by repeated measurements within the same analytical run, and inter-assay variability was assessed across different runs to ensure consistency of the biochemical analyses.

### 5.3. Histological and Immunohistochemical Evaluation

Achilles tendon samples collected for histological evaluation were embedded in hard paraffin using a paraffin embedding system (Leica Biosystems Inc., Leica Biosystems Nussloch GmbH, Nussloch, Germany) after routine tissue processing. Serial sections with a thickness of 4–5 µm were obtained. Immunohistochemical staining was performed using an automated IHC/ISH system (Bond Max, Leica Biosystems, Mount Waverley, Australia). Primary antibodies used for this procedure included VEGF(BT-APO9504, Bioassay Technology Laboratory, Shanghai, China), collagen type I (BT-APO2074, Bioassay Technology Laboratory, Shanghai, China), and collagen type III (BT-APO2081, Bioassay Technology Laboratory, Shanghai, China), followed by incubation with appropriate secondary antibodies.

The sections were evaluated by two independent histologists blinded to the study groups.

For each rat, 20 randomly selected microscopic fields from the tendon tissue sections were evaluated for histopathological damage on standard hematoxylin and eosin (H&E) stained slides using a semi-quantitative scoring system based on edema, inflammation, and hemorrhage. This approach is consistent with standard histopathological assessment practices in experimental studies, where multiple random fields are commonly evaluated to improve representativeness and reduce sampling bias [33,34]. All evaluations were performed under a light microscope at ×400 magnification. Each parameter was graded on a four-point ordinal scale: 0 = absent, 1 = mild involvement, 2 = moderate involvement, and 3 = severe involvement. The total histopathological damage score was calculated as the sum of the three parameters, yielding a possible score range of 0–9, where higher scores indicate more severe tissue damage.

In the immunohistochemical analyses, 15 randomly selected areas from each slide were evaluated for the presence and extent of immunopositive staining for vascular endothelial growth factor (VEGF), collagen type I, and collagen type III using a semi-quantitative scoring system. This general evaluation approach is commonly used in immunohistochemical studies [35,36,37]. For each primary antibody, a score of 0 was assigned when the finding involved less than 10% of the area, 1 point for 11–25%, 2 points for 26–50%, and 3 points for more than 50% involvement. Two blinded histopathologists independently evaluated all slides. To reduce observer-related bias, the final score for each sample was calculated as the average of the scores assigned by the two observers. It should be emphasized that this evaluation was semi-quantitative and based on the estimated percentage of immunopositive area, and that no quantitative intensity analysis (e.g., optical density or digital image analysis) was performed. Semi-quantitative scoring is inherently subject to observer-dependent subjective bias. Although two blinded histopathologists independently evaluated all slides and the mean of the two scores was used, this approach cannot completely eliminate inter-observer variability. Therefore, the immunohistochemical results should be regarded as relative, semi-quantitative estimates rather than absolute quantitative values.

The sample size for this study was calculated based on the method described by Charan et al., which considers the minimum and maximum values required for ANOVA statistical tests [38].

### 5.4. Statistical Analysis

All statistical analyses were performed using IBM SPSS Statistics version 18.0 (IBM Corp., Armonk, NY, USA). Data distribution was assessed using the Kolmogorov–Smirnov test together with visual inspection of histograms.

Continuous variables were expressed as mean ± standard deviation for normally distributed data and as median (interquartile range [IQR]) for non-normally distributed data.

For variables meeting the assumptions of normality and homogeneity of variance, comparisons among multiple groups were performed using one-way ANOVA. When significant differences were detected, pairwise comparisons were conducted using Bonferroni-adjusted post hoc tests. In cases where the assumption of homogeneity of variances was violated (based on Levene’s test), Tamhane’s T2 test was applied.

For non-normally distributed variables, comparisons among groups were performed using the Kruskal–Wallis test. When significant differences were identified, pairwise comparisons were performed using the Mann–Whitney U test with Bonferroni correction to control for multiple comparisons. The adjusted significance level was calculated based on the number of pairwise comparisons.

Biochemical parameters (TBARS and total thiol levels) were analyzed as continuous variables, whereas histopathological and immunohistochemical scores were analyzed using non-parametric methods due to their ordinal nature.

A two-tailed *p*-value of <0.05 was considered statistically significant.

## 6. Study Limitations and Strengths

This study has several limitations. First, the use of an animal model limits the generalizability of the findings to human tendon healing. Second, this study used only female Sprague–Dawley rats and did not consider the potential effect of sex on tendon healing. Estrogens and other hormones can influence inflammatory responses, collagen synthesis, and the healing process. To increase the generalizability of the results, future studies should include male animals [39]. Fosinopril was administered solely via the oral route; alternative dosages, durations, and local or intratendinous administration methods were not explored. Furthermore, fosinopril was evaluated at a single oral dose of 5 mg/kg/day, and no additional dose levels were included. Therefore, a dose–response relationship could not be established, and the effects of lower or higher doses on the biochemical and histological outcomes remain unknown. Future studies incorporating multiple dose levels are needed to characterize the dose–response profile and determine an optimal dosing range for tendon healing. Moreover, the lack of biomechanical evaluation prevented assessment of the functional durability of the healed tendon. Finally, the study focused only on short-term effects and did not provide data on long-term outcomes or sustained healing. These limitations highlight the need for future studies with broader scope, longer follow-up periods, and varied dosing protocols, both experimental and clinical in nature. Furthermore, the immunohistochemical analyses were semi-quantitative and based on visual estimation of the percentage of immunopositive areas, without quantitative intensity measurement or digital image analysis. This method is inherently susceptible to observer-related subjective bias, and future studies using quantitative or automated image analysis are needed to confirm our findings.

Furthermore, since TBARS and total thiol measurements were performed only in serum, these findings reflect systemic redox status and cannot be interpreted as direct evidence of oxidative changes at the Achilles tendon injury site. Tendon tissue-specific TBARS and total thiol levels were not assessed in the present study; therefore, future studies incorporating local tendon tissue-specific oxidative stress and antioxidant measurements are warranted.

The Achilles tendon is one of the most frequently injured and rupture-prone tendons in the body. Such injuries are particularly common in middle-aged and older populations. Given the high prevalence of hypertension in these age groups, investigating the potential effects of ACE inhibitors—commonly used in hypertension treatment—on tendon healing is of notable clinical relevance. Our literature review revealed no prior study that directly examined the effect of fosinopril on tendon healing, underscoring the originality of our study and its contribution to the existing body of knowledge. Finally, it should be noted that this study mainly assesses tendon healing from biochemical and histological perspectives and does not include biomechanical tests to evaluate functional recovery. Accordingly, our results should be interpreted as phenotypic effects compatible with ACE inhibition, rather than definitive mechanistic evidence. Future studies incorporating direct measurements of ACE activity, angiotensin II levels, and related signaling pathways (e.g., TGF-β) are required to confirm the underlying molecular mechanisms. Previous studies have emphasized the biological and biomechanical aspects of tendon healing, including tissue organization and functional recovery processes [40,41], supporting the need for comprehensive biomechanical and functional assessments in future research to better characterize tendon healing outcomes.

## 7. Conclusions

This experimental study is one of the first to evaluate the effects of the ACE inhibitor fosinopril on the healing process following surgical repair of Achilles tendon rupture. Biochemical analyses showed lower serum TBARS levels and higher total thiol levels in the fosinopril-treated groups, indicating a shift toward a more favorable systemic redox profile. Histopathological evaluation demonstrated reduced edema, inflammation, and hemorrhage in the repair + fosinopril groups compared with the repair-only groups. In parallel, immunohistochemical analyses showed significantly lower expression of VEGF, collagen type I, and collagen type III in the repair + fosinopril groups. Taken together, these findings demonstrate that fosinopril administration was associated with reduced systemic oxidative stress markers, enhanced antioxidant capacity, lower histopathological damage scores, and modulation of angiogenic and extracellular matrix-related markers during the evaluated period of tendon healing.

Based on these combined biochemical, histopathological, and immunohistochemical findings, we hypothesize that fosinopril may modulate the tissue response to tendon injury and potentially limit excessive scar formation during tendon repair. However, this proposed effect on scar formation remains a hypothesis and cannot be confirmed by the present study, as scar formation was not directly quantified and biomechanical and functional outcomes were not assessed. Further studies incorporating direct quantitative assessment of scar formation, biomechanical and functional outcomes, longer follow-up periods, and mechanistic analyses are required to verify this hypothesis.

## Figures and Tables

**Figure 1:**
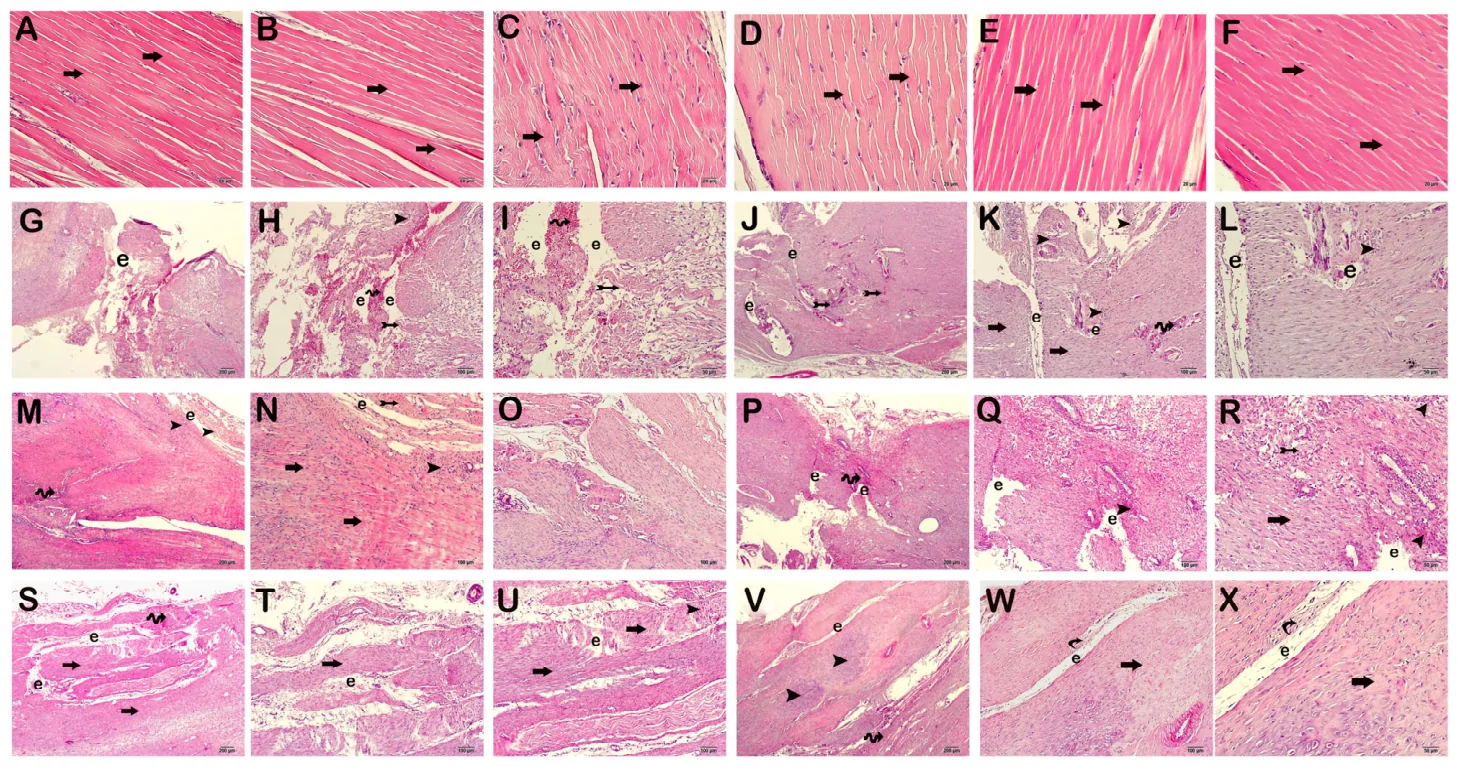
Histopathological evaluation of tendon healing (H&E staining). Representative images of Achilles tendon sections from different experimental groups at Days 7, 14, and 28. (**A**–**C**) Healthy control group showing normal tendon architecture with parallel, well-organized collagen fibers. (**D**–**F**) Fosinopril-only group demonstrating preserved tendon structure similar to controls. (**G**–**I**) Repair-only group (Day 7) showing marked edema, inflammatory cell infiltration, hemorrhage, and disorganized collagen fibers. (**J**–**L**) Repair-only group (Day 14) with reduced edema and inflammation but persistent structural disruption. (**M**–**O**) Repair-only group (Day 28) showing partial recovery with residual disorganization. (**P**–**R**) Repair + fosinopril group (Day 7) demonstrating reduced inflammation and edema compared to repair-only. (**S**–**U**) Repair + fosinopril group (Day 14) with further reduction in inflammatory changes and improved collagen organization. (**V**–**X**) Repair + fosinopril group (Day 28) showing well-organized, parallel collagen fibers and minimal inflammatory findings. Symbols: Arrows indicate normal collagen fibrils. Arrowheads indicate polymorphonuclear leukocyte (PMNL) infiltration. Spiral arrows indicate hemorrhagic areas. Tailed arrow indicates degenerative collagen fibrils. Letter “e” indicates edematous regions. Curved arrows indicate newly synthesized and organized collagen fibrils. Magnifications: ×4, ×20, ×40 as indicated. HHS: Histopathological Healing Score.

**Figure 2:**
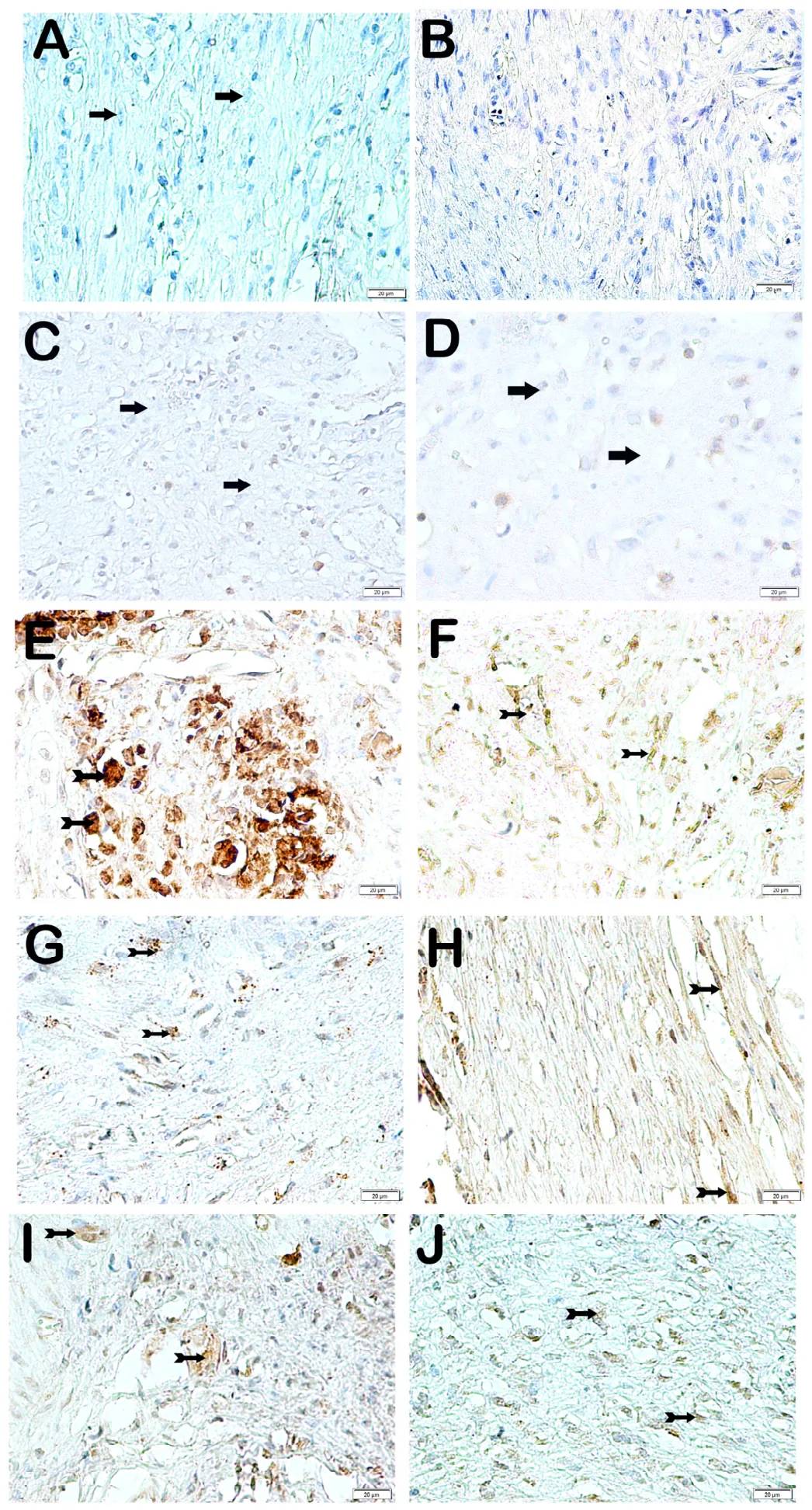
Immunohistochemical staining for collagen type I. Representative images showing collagen type I expression in Achilles tendon tissue across experimental groups. (**A**) Healthy control group showing absence of collagen I immunoreactivity. (**B**–**D**) Fosinopril-only groups (Days 7, 14, 28) showing minimal or absent staining. (**E**,**F**) Repair-only groups (Days 7 and 14) demonstrating strong collagen I positivity. (**G**) Repair-only group (Day 28) showing moderate collagen I expression. (**H**–**J**) Repair + fosinopril groups (Days 7, 14, 28) demonstrating progressively reduced collagen I expression compared to repair-only groups. Collagen I expression is presented as semi-quantitative immunoreactivity. Arrows indicate immunonegative tendinocytes. Tailed Arrows indicate collagen I-positive cells. Magnification: ×40.

**Figure 3:**
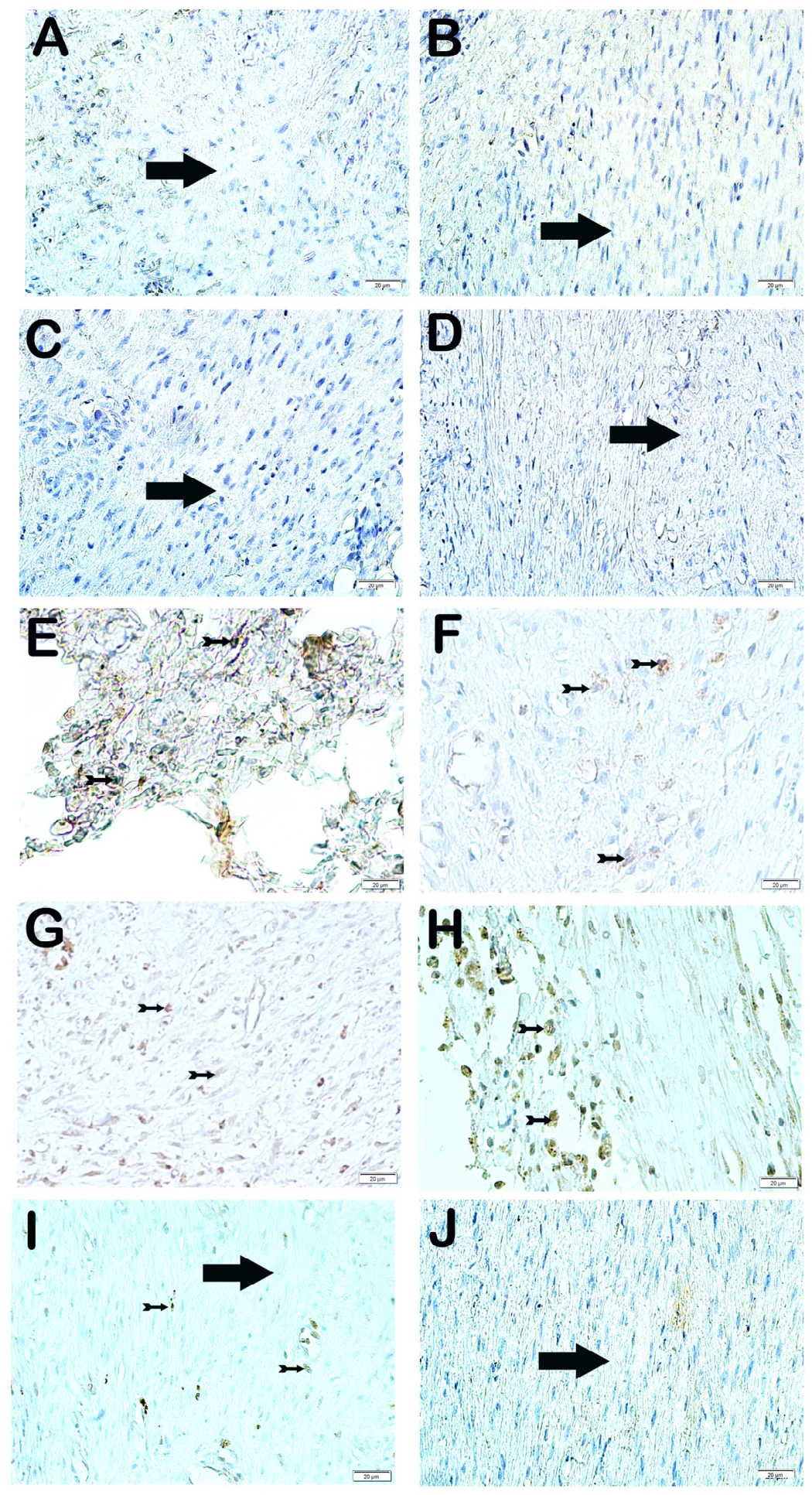
Immunohistochemical staining for collagen type III. Representative images showing collagen type III expression in Achilles tendon tissue. (**A**) Healthy control group demonstrating absence of collagen III immunoreactivity. (**B**–**D**) Fosinopril-only groups (Days 7, 14, 28) showing minimal or no staining. (**E**,**F**) Repair group (Days 7 and 14) demonstrating strong collagen III positivity. (**G**) Repair group (Day 28) showing moderate expression. (**H**–**J**) Repair + fosinopril groups (Days 7, 14, 28) demonstrating reduced collagen III expression compared to the repair group. Arrows indicate collagen III-negative tendinocytes. Tailed arrows indicate collagen III-positive areas. Magnification: ×40.

**Figure 4:**
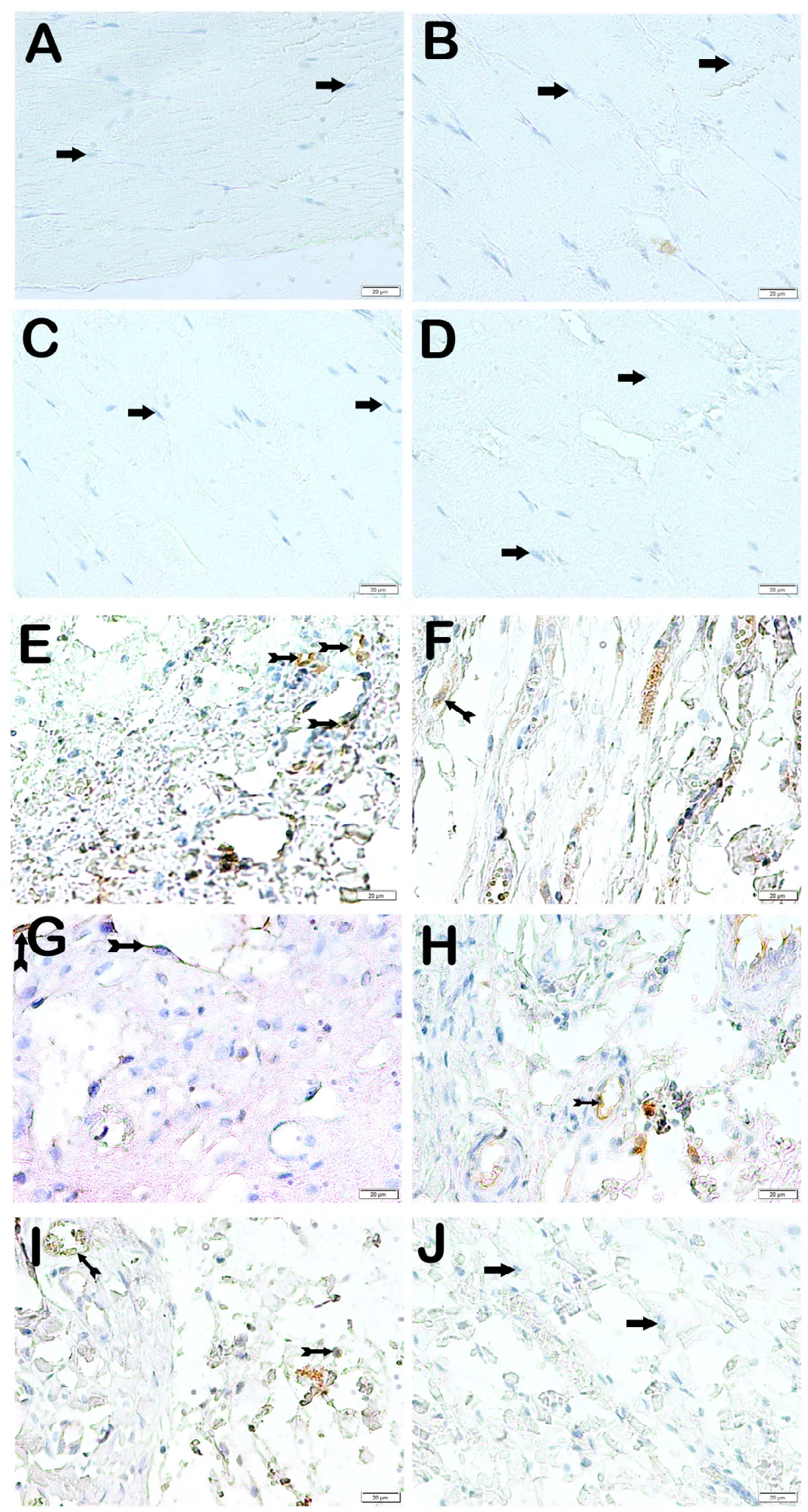
Immunohistochemical staining for VEGF. Representative images of VEGF expression in Achilles tendon sections. (**A**) Healthy control group showing absence of VEGF staining. (**B**–**D**) Fosinopril-only groups (Days 7, 14, 28) demonstrating minimal VEGF expression. (**E**,**F**) Repair group (Days 7 and 14) showing strong VEGF positivity, indicating active angiogenesis. (**G**) Repair group (Day 28) showing moderate VEGF expression. (**H**–**J**) Repair + fosinopril groups (Days 7, 14, 28) demonstrating reduced VEGF expression compared to the repair group. Arrows indicate VEGF-negative tendinocytes. Tailed arrows indicate VEGF-positive staining areas. Magnification: ×40.

**Figure 5:**
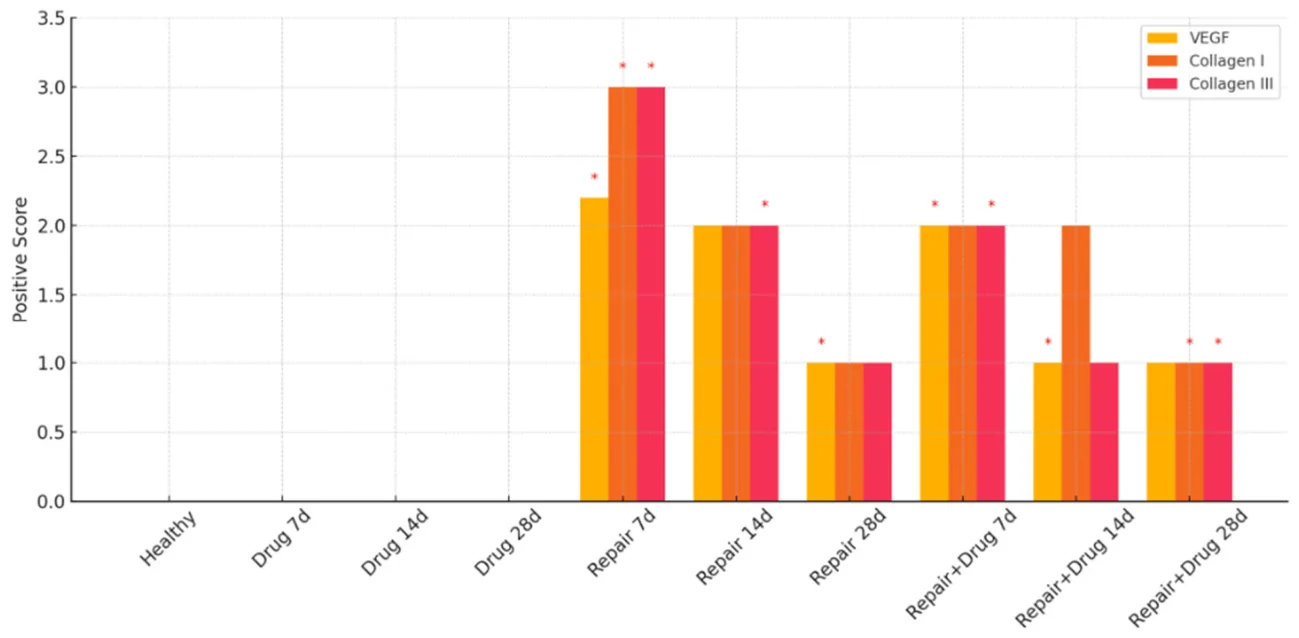
Semi-quantitative Analysis Results (*: *p* < 0.05).

**Figure 6:**
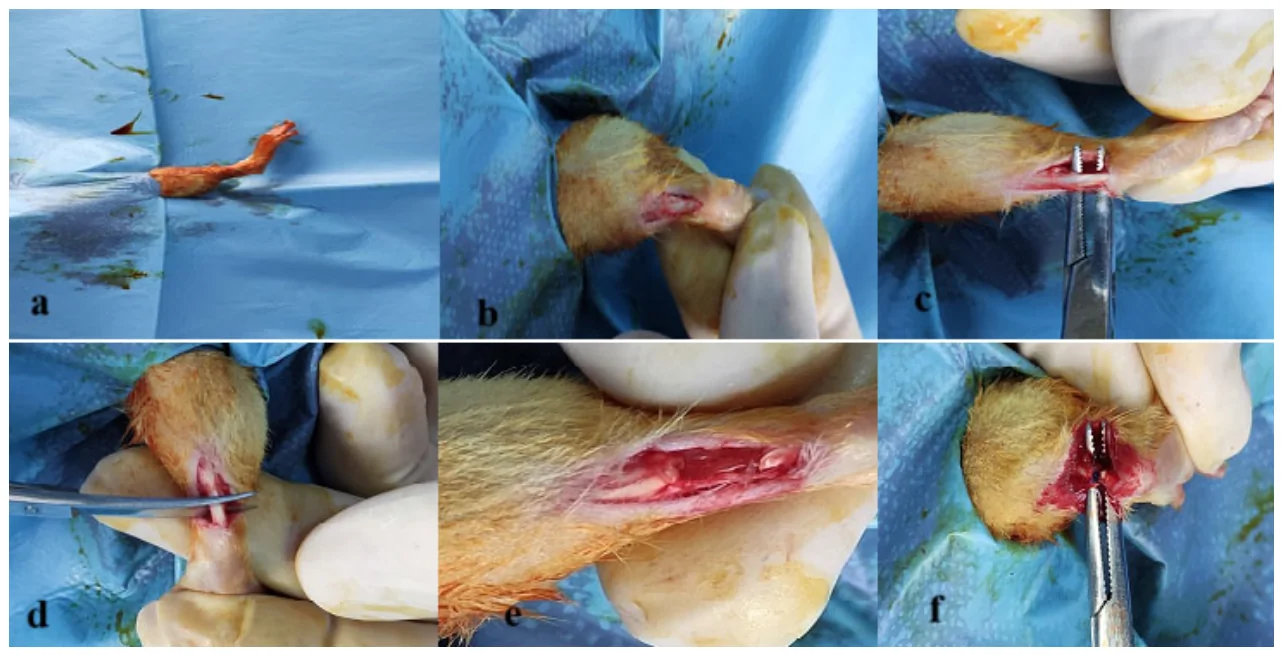
(**a**) Preparation of the surgical field. (**b**) Skin incision to access the tendon. (**c**) Exposure of the Achilles tendon through blunt dissection. (**d**) Transverse transection of the Achilles tendon. (**e**) Appearance of the ruptured Achilles tendon. (**f**) Surgical repair of the Achilles tendon using the modified Kessler technique.

**Table 1:** Serum TBARS levels (nmol/mL) in study groups at different time points.

Group	Day 7 (Mean ± SD)	Day 14 (Mean ± SD)	Day 28 (Mean ± SD)
Healthy Control	–	–	7.14 ± 0.55 a *
Repair-only	14.28 ± 7	8.48 ± 1.47 a *	7.66 ± 1.38 a *
Repair + Fosinopril	9.20 ± 2.37 a *	7.93 ± 1.03 a *	7.07 ± 1.12 a *
Drug-only (Fosinopril)	8.67 ± 2.41 a *	8.60 ± 1.46 a *	6.54 ± 1.14 a *

* *p* < 0.05 vs. Repair Day 7. a: vs. Repair-only. Values are presented as mean ± standard deviation. Mann–Whitney U test with Bonferroni correction was used for group comparisons. Significant differences (*p* < 0.05) are indicated compared with the Repair—Day 7 group.

**Table 2:** Serum total thiol levels (µmol/L) in study groups at different time points.

Groups	Day 7 (Median [IQR])	Day 14 (Median [IQR])	Day 28 (Median [IQR])
Healthy Control	–	–	288 (55) b, c, d
Repair-only	198 (82) a, c, d	186 (135) a, b, c, d	206 (37) a, d
Repair + Fosinopril	206 (100) a, b, d	212 (48) a, b, c, d	244 (125) a, d
Drug-only (Fosinopril)	226 (122) a, b, c, d	246 (183) b, c, d	360 (114) a

Symbols indicate statistically significant pairwise differences (*p* < 0.05) as follows: a, vs. Healthy Control; b, vs. Repair—Day 28; c, vs. Repair + Fosinopril—Day 28; d, vs. Fosinopril only—Day 28. Pairwise comparisons were performed using the Mann–Whitney U test with Bonferroni correction. Values are presented as median (interquartile range).

**Table 3:** Histopathological Damage Score (HDS) Results.

Groups	Edema (Median [Range])	Inflammation (Median [Range])	Hemorrhage (Median [Range])	HHS
Healthy Control Group	0 (0–0)	0 (0–0)	0 (0–0)	0 (0–1)
Drug Only—Day 7	0 (0–0)	0 (0–0)	0 (0–0)	0.5 (0–1)
Drug Only—Day 14	0 (0–0)	0 (0–0)	0 (0–0)	0 (0–1)
Drug Only—Day 28	0 (0–1)	0 (0–1)	0 (0–0)	0 (0–1)
Repair—Day 7	3 (3–3)	1 (1–2)	2 (2–3)	6.5 (5–7)
Repair—Day 14	2 (2–2)	2 (2–3)	2 (1–2)	5.5 (5–6)
Repair—Day 28	2 (1–2)	2 (2–2)	1 (1–1)	5 (4–5.5)
Repair + Drug—Day 7	2 (2–2)	1 (0–1)	1 (1–1)	4 (3–4)
Repair + Drug—Day 14	2 (1–1)	1 (1–2)	0 (0–1)	4 (3–4)
Repair + Drug—Day 28	1 (1–2)	1 (1–1)	0 (0–0.5)	2 (2–3)

**Table 4:** Semi-quantitative Analysis Results.

Groups	VEGF Positivity Score (Median [Range])	Collagen I Positivity Score (Median [Range])	Collagen III Positivity Score (Median [Range])
Healthy Control Group	0 (0–0)	0 (0–0)	0 (0–0)
Drug Only—Day 7	0 (0–0)	0 (0–0)	0 (0–0)
Drug Only—Day 14	0 (0–0.5)	0 (0–0)	0 (0–0)
Drug Only—Day 28	0 (0–0)	0 (0–0)	0 (0–0)
Repair—Day 7	2 (2–3)	3 (2.5–3)	3 (2–3)
Repair—Day 14	2 (1–2)	2 (2–2.5)	2 (2–2)
Repair—Day 28	1.5 (1–2)	2 (2–2)	2 (2–2)
Repair + Drug—Day 7	1 (1–1)	2 (2–2)	2 (2–2)
Repair + Drug—Day 14	0 (0–1)	1 (1–2)	1 (1–2)
Repair + Drug—Day 28	0 (0–1)	0 (0–1)	0 (0–1)

**Table 5:** Study Groups and Sacrifice Time Points.

Main Group	Subgroup	Intervention	Sacrifice Time Point	Number of Rats (*n*)
**Group 1**	1a	No intervention performed	Week 4	8
**Group 2**	2a	Achilles tendon rupture + surgical repair	Week 1	8
	2b	Achilles tendon rupture + surgical repair	Week 2	8
	2c	Achilles tendon rupture + surgical repair	Week 4	8
**Group 3**	3a	Achilles tendon rupture + surgical repair + fosinopril	Week 1	8
	3b	Achilles tendon rupture + surgical repair + fosinopril	Week 2	8
	3c	Achilles tendon rupture + surgical repair + fosinopril	Week 4	8
**Group 4**	4a	Fosinopril administration only	Week 1	8
	4b	Fosinopril administration only	Week 2	8
	4c	Fosinopril administration only	Week 4	8

## Data Availability

All data generated for the manuscript are included in the study and are available upon request.

## Abbreviations

ACE	Angiotensin converting enzyme
TBARS	Thiobarbituric acid reactive substances
VEGF	Vascular endothelial growth factor
PMNL	Polymorphonuclear leukocyte
H&E	Hematoxylin and eosin
